# A novel, species-specific, real-time PCR assay for the detection of the emerging zoonotic parasite *Ancylostoma ceylanicum* in human stool

**DOI:** 10.1371/journal.pntd.0005734

**Published:** 2017-07-10

**Authors:** Marina Papaiakovou, Nils Pilotte, Jessica R. Grant, Rebecca J. Traub, Stacey Llewellyn, James S. McCarthy, Alejandro J. Krolewiecki, Rubén Cimino, Rojelio Mejia, Steven A. Williams

**Affiliations:** 1 Department of Biological Sciences, Smith College, Northampton, Massachusetts, United States of America; 2 Molecular and Cellular Biology Program, University of Massachusetts, Amherst, Massachusetts, United States of America; 3 Faculty of Veterinary and Agricultural Science, University of Melbourne, Parkville, Australia; 4 Clinical Medical Laboratory, QIMR Berghofer Medical Research Institute, Herston, Queensland, Australia; 5 Instituto de Investigaciones en Enfermedades Tropicales, Universidad Nacional de Salta/CONICET, Orán, Argentina; 6 Instituto de Patología Experimental (IPE-CONICET), Facultad de Ciencias de la Salud, Universidad Nacional de Salta, Salta, Argentina; 7 National School of Tropical Medicine, Baylor College of Medicine, Houston, Texas, United States of America; George Washington University, UNITED STATES

## Abstract

**Background:**

Molecular-based surveys have indicated that *Ancylostoma ceylanicum*, a zoonotic hookworm, is likely the second most prevalent hookworm species infecting humans in Asia. Most current PCR-based diagnostic options for the detection of *Ancylostoma* species target the Internal Transcribed Spacer (ITS) regions of the ribosomal gene cluster. These regions possess a considerable degree of conservation among the species of this genus and this conservation can lead to the misidentification of infecting species or require additional labor for accurate species-level determination. We have developed a novel, real-time PCR-based assay for the sensitive and species-specific detection of *A*. *ceylanicum* that targets a non-coding, highly repetitive genomic DNA element. Comparative testing of this PCR assay with an assay that targets ITS sequences was conducted on field-collected samples from Argentina and Timor-Leste to provide further evidence of the sensitivity and species-specificity of this assay.

**Methods/Principal findings:**

A previously described platform for the design of primers/probe targeting non-coding highly repetitive regions was used for the development of this novel assay. The assay’s limits of detection (sensitivity) and cross-reactivity with other soil-transmitted helminth species (specificity) were assessed with real-time PCR experiments. The assay was successfully used to identify infections caused by *A*. *ceylanicum* that were previously only identified to the genus level as *Ancylostoma* spp. when analyzed using other published primer-probe pairings. Further proof of sensitive, species-specific detection was provided using a published, semi-nested restriction fragment length polymorphism-PCR assay that differentiates between *Ancylostoma* species.

**Conclusions/Significance:**

Due to the close proximity of people and domestic/wild animals in many regions of the world, the potential for zoonotic infections is substantial. Sensitive tools enabling the screening for different soil-transmitted helminth infections are essential to the success of mass deworming efforts and facilitate the appropriate interpretation of data. This study describes a novel, species-specific, real-time PCR-based assay for the detection of *A*. *ceylanicum* that will help to address the need for such tools in integrated STH deworming programs.

**Trial registration:**

ANZCTR.org.au ACTRN12614000680662

## Author summary

Historically, *Ancylostoma ceylanicum* has been viewed as an uncommon cause of human hookworm infection, with minimal public health importance. However, recent reports have indicated that this zoonotic hookworm causes a much greater incidence of infection within certain human populations than was previously believed. Current methods for the species-level detection of *A*. *ceylanicum* rely on techniques that involve conventional PCR accompanied by restriction enzyme digestions. These PCR-based assays are not only laborious but they lack sensitivity as they target suboptimal regions on the DNA. As efforts aimed at the eradication of hookworm disease have grown substantially over the last decade, the need for sensitive and specific tools to monitor and evaluate programmatic successes has correspondingly escalated. Since a growing body of evidence suggests that patient responses to drug treatment can vary based upon the species of hookworm that is causing infection, accurate species-level diagnostics are advantageous. Accordingly, the novel real-time PCR-based assay described here provides a sensitive, species-specific diagnostic tool that will facilitate the accurate mapping of disease endemicity and will aid in the evaluation of progress of programmatic deworming efforts.

## Introduction

*Ancylostoma ceylanicum* is the only predominantly animal-infecting hookworm species known to successfully develop into adults within the human intestine [1]. This zoonotic species has been reported to infect animals and humans in Southeast Asia, South America, South Africa, Melanesia and Australia [2–13], and prevalence studies have demonstrated *A*. *ceylanicum* to be the second most common hookworm species causing human infection in many parts of Asia [5, 14–16]. Equally troubling, a recent study investigating *A*. *ceylanicum* transmission conducted in Cambodia demonstrated the ability of this worm to result in burdens of infection comparable to levels caused by *Necator americanus* [6]. Furthermore, it has been demonstrated that *Ancylostoma duodenale* can ingest up to eight times the volume of blood consumed by *N*. *americanus*, resulting in increased iron depletion [17]. Accordingly, frequent cases of *Ancylostoma*-associated anemia have been reported, even among properly nourished individuals [12, 18]. Although only speculative, similar pathology might exist for human infections with *A*. *ceylanicum*, underscoring the potential importance of this zoonotic parasite for both child and maternal health [19]. Despite these findings, methodologies for the reliable, species-specific, and sensitive molecular detection of *A*. *ceylanicum* are lacking, resulting in a need for improved diagnostic tools.

Historically, diagnosis and detection of all hookworm species has relied heavily on the use of coprological methods [5]. Widespread use of these methods stems from their low cost, simplicity, and minimal infrastructure requirements. By default, microscopy has served as the standard method for gastrointestinal helminth detection [5]. However, at the egg level, and in some cases even at the larval level, certain hookworm species are morphologically indistinguishable [20] and further culturing of worms is required for differentiation. Conducting such coprological techniques is time-consuming and requires highly skilled microscopists for accurate assessment of results [21]. Accordingly, PCR-based molecular diagnostic tests for hookworm provide an attractive alternative to microscopy with more modest training requirements. However, such assays frequently target the internal transcribed spacer regions (ITS) of the rDNA gene clusters, which demonstrate considerable homology between closely related *Ancylostoma* species [5]. This similarity can lead to species misidentification in hookworm positive samples. Several PCR variations such as nested PCR and PCR coupled with restriction fragment length polymorphism (PCR-RFLP) have been developed to differentiate between the hookworm species. Unfortunately, these methods tend to be laborious, requiring many steps and/or restriction enzyme digestions coupled with agarose gel electrophoresis [4, 21, 23, 24]. In addition, nested PCR is far more likely to result in false positivity due to sample contamination and increased numbers of PCR cycles [22]. Thus, simplified PCR assays, not reliant on nested PCR or restriction enzyme digestions, would be of great benefit to programmatic efforts. Furthermore, while drug intervention and hygiene programs have made substantial gains and continue to show great promise, they frequently fail to account for the control of zoonotic infections [15]. Such concerns are particularly important when proper sanitation is lacking as the potential for zoonotic transmission from wild or domestic animals to humans is dramatically increased [5]. Accordingly, species-level identification provides insight into possible infection reservoirs, which might not be considered utilizing genus-level diagnostics.

Herein we report the development and validation of a novel, real-time PCR-based diagnostic assay for the species-specific detection of *A*. *ceylanicum*. The development of this test is based on the identification of a highly repetitive, non-coding DNA element using advanced bioinformatics to analyze whole genome sequence data for target discovery and assay design [25]. We posit that the incorporation of this new assay into our previously established diagnostic platform [25] will aid programmatic efforts, as accurate species-level detection and differentiation of *Ancylostoma* ssp. is now possible.

## Materials and methods

### Assay design

Genome sequences for *A*. *ceylanicum* were obtained from NCBI (Sequence Read Archive ID: SRR2037027 and SRR2037046). Analysis of repeat DNA content was performed using the publicly available platform, RepeatExplorer [26], following our previously established bioinformatics workflow [25], and a primer-probe set was designed using the PrimerQuest online tool (Integrated DNA Technologies, Coralville, IA) (Table 1). This candidate primer-probe pairing then underwent further bioinformatics analysis using the Primer-BLAST tool available from the National Center for Biotechnology Information (NCBI) website. Utilizing default parameters, the candidate primer pair did not return any matches to off-target templates within NCBI’s Nucleotide Collection database, indicating target specificity and ensuring that the occurrence of off-target amplification would be extremely unlikely. Default parameters for probe-based PCR were utilized for the assay design and the probe was labeled with 6FAM fluorophore at the 5’ end and double-quenched with ZEN (internal) and 3IABkFQ at the 3’ end.

**Table 1:** Primer and probe sequences for *A*. *ceylanicum*.

Parasite	Forward	Reverse	Probe
*A. ceylanicum*	5’-CAAATATTACTGTGCGCATTTAGC-3’	5’-GCGAATATTTAGTGGGTTTACTGG-3’	5’-/56-FAM CGGTGAAAG/ZEN/CTTTGCGTTATTGCGA/3IABkFQ/ -3’

### Assay optimization

A concentration matrix was created to determine the optimal forward and reverse primer concentration as previously described [25]. All reactions were performed in 7 μl total volumes utilizing 2 μl of template, 3.5 μl of TaqMan Fast Universal PCR Master Mix (ThermoFisher Scientific, Waltham, MA), and the appropriate concentrations of primers and probe. Cycling conditions consisted of an initial 2-minute incubation step at 50°C, followed by a 10-minute incubation at 95°C. These incubations were followed by 40 cycles of 95°C for 15 seconds for denaturation and 1 minute at 59°C for primer annealing and polymerase extension. All reactions were conducted using the StepOne Plus Real-Time PCR System (Life Technologies, Carlsbad, CA).

### Sensitivity and specificity testing

Following the completion of primer optimization reactions, the sensitivity and specificity of our assay was evaluated. For sensitivity testing, the optimized assay was performed utilizing a titration of genomic DNA concentrations as template. Reactions were conducted using 2 μl of *A*. *ceylanicum* genomic template at concentrations of 1 ng/μl, 100 pg/μl, 10 pg/μl, 1 pg/μl, 100 fg/μl, 10 fg/μl, 1 fg/μl, 100 ag/μl 10 ag/μl, and 1 ag/μl. In order to verify assay specificity, optimized conditions were employed to evaluate the potential for non-specific amplification of control genomic DNA from six other species of soil-transmitted helminths (*A*. *duodenale*, *N*. *americanus*, *Trichuris trichiura*, *Strongyloides stercoralis*, *Ascaris lumbricoides and Ancylostoma caninum*), along with human genomic DNA and DNA of the common gastrointestinal tract commensal bacterium *Escherichia coli* (strain K-12). All of these control samples were tested utilizing 2 μl of DNA template at concentrations of 1 ng/μl.

### Mixed infection testing

A panel of six 10-fold serial dilutions of an initial 1 ng/μl stock of *A*. *ceylanicu*m genomic DNA was created. To each dilution in this panel, genomic DNA was added from both *A*. *caninum* and *A*. *duodenale* such that the concentrations of DNA from both of these other species were at a final concentration of 1 ng/μl in each panel dilution. The aforementioned panel was tested with the optimized real-time PCR based assay for *A*. *ceylanicum* to prove that the presence of other *Ancylostoma* spp., simulating mixed infections, would not affect our assay’s specificity. Furthermore, testing of this panel enabled us to determine the assay’s limits of detection when increasingly limited concentrations of target DNA (*A*. *ceylanicum*) were intermixed with genomic DNA from the two other *Ancylostoma* spp. (S1 Table).

### Selection of field-samples for inclusion in this study

Sixty-one human stool samples previously collected as part of the “Wash for Worms” intervention trial in Timor-Leste (Trial registration: ACTRN12614000680662) were selected for inclusion in this study. DNA extractions for these samples were previously performed at QIMR Berghofer, and real-time PCR analysis demonstrated the presence of *Ancylostoma* spp. DNA in 22 of these samples [27]. Eight additional samples, collected in Orán, Argentina, as part of a larger collection effort (IRB00008019), were also selected for inclusion in this study. For Argentinian samples, DNA was previously extracted at Baylor College of Medicine utilizing a published methodology [28]. Prior real-time PCR analysis of these eight samples had demonstrated the presence of *Ancylostoma* ssp. DNA [28].

### PCR-RFLP analysis

A previously published semi-nested conventional PCR assay coupled with restriction fragment length polymorphism analysis [23] was employed to distinguish between the different hookworm species in the samples obtained from both Timor-Leste and Orán, Argentina. All amplification reactions and digestions were conducted in accordance with the previously described methodologies [23].

### Real-time PCR analysis

DNA aliquots of all samples from both Timor-Leste and Orán, Argentina were sent to Smith College for further analysis. Samples were blindly coded and assayed for the presence of both *A*. *duodenale* and *A*. *ceylanicum*. Testing for the presence of *A*. *duodenale* occurred in accordance with the previously described protocol [25], while *A*. *ceylanicum* testing was conducted using the novel assay described here.

## Results

Utilization of the previously published workflow for repeat analysis led to the identification of a novel target for the sensitive and species-specific real-time PCR-based detection of *A*. *ceylanicum* [25]. Employing the PrimerQuest online tool, a primer/probe pairing was identified and oligonucleotides were synthesized. Through the analysis of a titration matrix, primer optimization reactions were performed, and the optimal forward primer concentration was determined to be 125 nM, while the reverse primer was demonstrated to have an optimal concentration of 1000 nM. This combination of concentrations resulted in the lowest Ct values when amplifying 2 μl of *A*. *ceylanicum* genomic DNA at a concentration of 1 ng/μl.

Assay specificity testing failed to amplify purified genomic DNA from the STH parasites *A*. *duodenale*, *N*. *americanus*, *T*. *trichiura*, *S*. *stercoralis*, *A*. *lumbricoides and A*. *caninum*. Testing also failed to amplify human DNA or DNA from the common gastrointestinal bacteria, *E*. *coli* (strain K-12), thus demonstrating the assay’s species-specific detection properties. Furthermore, assay sensitivity testing demonstrated consistent detection of purified *A*. *ceylanicum* genomic DNA template at all quantities above 200 ag. However, when the *A*. *ceylanicum* assay was utilized to analyze samples containing simulated mixed infections of both *A*. *ceylanicum* and other *Ancylostoma* species, the limit of detection was determined to be 13.3 fg of *A*. *ceylanicum* DNA (S1 Table). Importantly, based upon the genome size of *A*. *ceylanicum* [29], and assuming that a single egg contains approximately 8 cells, this quantity of template DNA is less than the quantity expected to be found within a single *A*. *ceylanicum* egg (theoretically 5520 fg of DNA based on *A*. *ceylanicum*’s genome size).

To further validate this novel assay, 61 DNA extracts from stool samples collected in Timor-Leste were analyzed. Utilizing a genus-specific real-time PCR assay, previous testing conducted at QIMR Berghofer demonstrated the presence of *Ancylostoma* ssp. DNA in 22 of these samples [27]. However, follow-up testing of these samples utilizing our previously described *A*. *duodenale*-specific primer/probe set excluded the presence of *A*. *duodenale* [25], and semi-nested PCR-RFLP analysis demonstrated the presence of *A*. *ceylanicum* in 21 of the 22 samples analyzed [25]. The identity of the 22^nd^ sample could not be determined as this sample failed to amplify using both our *A*. *duodenale-*specific assays and the semi-nested PCR-RFLP analysis. Two independent sequencing trials were also performed, but meaningful results could not be obtained. These experiments exhausted the sample stock and so no further experiments could be done to identify this sample. This subset of 22 *Ancylostoma* ssp.-positive samples was then employed to demonstrate the comparative sensitivity and species-specificity of our newly designed *A*. *ceylanicum* assay. Utilizing this assay, the same 21 samples which were determined to be *A*. *ceylanicum-*positive by RFLP-PCR again tested positive for the presence of *A*. *ceylanicum* DNA, while the 22^nd^ sample (PCR-RFLP-negative for *A*. *ceylanicum*) was also negative by real-time PCR, likely indicating that the *Ancylostoma* genus-specific assay (QIMR) had amplified a non-*duodenale*, non-*ceylanicum Ancylostoma* target (S2 Table). Equally important, the remaining 39 Timor-Leste-derived samples that tested negative for *Ancylostoma* spp. using the previously described genus-specific PCR assay [27] also produced a negative result when tested using the newly described *A*. *ceylanicum*-specific diagnostic (S2 Table). Thus, negative results agreed across all samples, in all replicates, with all utilized assays.

While testing of field-collected samples from Timor-Leste demonstrated the ability of our new assay to detect DNA from *A*. *ceylanicum*, we next sought to demonstrate that this *A*. *ceylanicum* real-time PCR assay would not amplify DNA from *A*. *duodenale*-containing field samples. For this purpose, eight additional DNA extracts from stool samples, obtained from an *A*. *duodenale* endemic region near Orán, Argentina, were tested in duplicate. Utilizing a combination of the previously described *A*. *duodenale*-specific PCR [25] and the semi-nested PCR-RFLP assays [23], we verified the presence of *A*. *duodenale* and the absence of *A*. *ceylanicum* within each sample (S1 Fig, S3 Table). Individual samples were then tested using the newly described *A*. *ceylanicum*-specific PCR assay, and for all samples, in all replicates, amplification failed to occur. Thus, by selectively amplifying *A*. *ceylanicum*-containing samples from Timor-Leste, but failing to amplify Argentinian samples positive for *A*. *duodenale*, we successfully demonstrated the species-specific nature of the *A*. *ceylanicum* PCR assay on human samples collected in the field. Table 2 summarizes the results of the various molecular approaches used to determine the species of *Ancylostoma* within samples from both field studies.

**Table 2:** Results of three separate molecular assays for *Ancylostoma* spp. Differentiation.

Origin of samples	Real-time PCR for *A*. *duodenale* [25]	Real-time PCR for *A*. *ceylanicum*	Semi-nested PCR [23]
**Timor-Leste**	(-)	(+)*	(Fig 1)
**Orán, Argentina**	(+)	(-)	(S1 Fig)

*Twenty-one out of twenty-two samples were positive for *A*. *ceylanicum*. The identity of the species in the 22^nd^ sample could not be determined utilizing any of the employed PCR assays or Sanger sequencing

**Fig 1:**
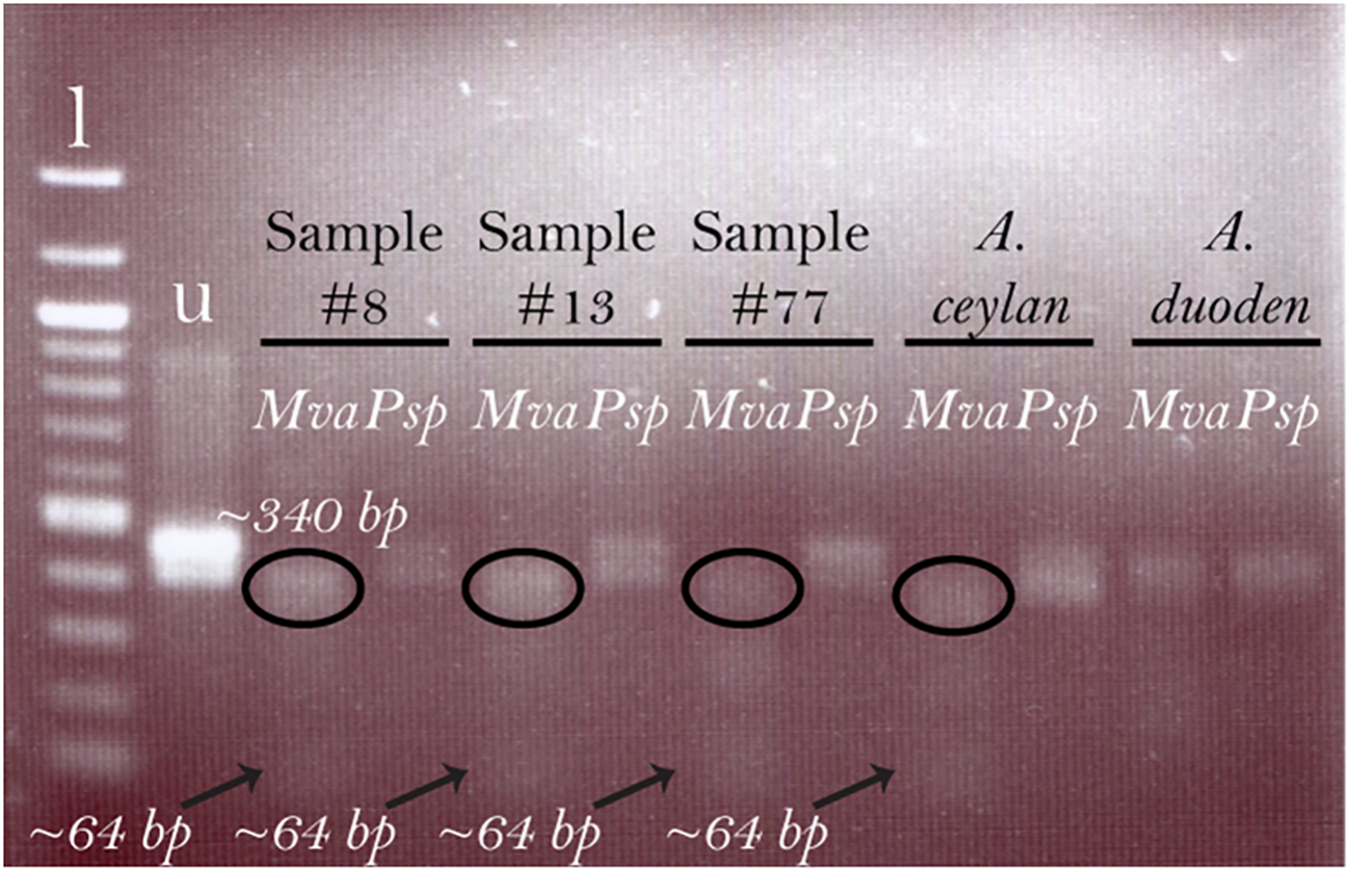
*Ancylostoma* spp. determination using semi-nested PCR-RFLP analysis. Following two rounds of conventional PCR targeting the ITS region, the product (~ 400 bp) was subjected to two separate restriction enzyme digestions (MvaI/BstN1 [#ER0551] and Psp1406I/AcII [#ER0942], ThermoFisher Scientific) following the manufacturer’s suggested protocol. The MvaI enzyme digests PCR amplicons of *A*. *ceylanicum* into two products (bands at 340 bp and 64 bp) but does not digest amplicons of *A*. *caninu*m or *A*. *duodenale*. Psp1406I digests *A*. *duodenale* amplicons into two products (bands at 255 bp and 149 bp) but does not digest amplicons of *A*. *caninum* or *A*. *ceylanicum*. In Fig 1, the uncut product (2^nd^ round PCR product), the Mva product (2^nd^ round PCR product digested with MvaI) and the Psp product (2^nd^ round PCR product digested with Psp1 enzyme) for the positive control (*A*. *ceylanicum*) and for samples from Timor-Leste are shown. The banding pattern demonstrates the presence of *A*. *ceylanicum* in these samples and validates the positive results from the newly described real-time PCR assay for *A*. *ceylanicum* (S2 Table; l = 100 base pair ladder; u = undigested control).

## Discussion

Highlighting the under-recognized importance of *A*. *ceylanicum* as a public health concern, a study in northern Cambodia recently implicated *A*. *ceylanicum* as the causative agent responsible for as many as half of all human hookworm infections among the young adult population [6]. However, despite such prevalence, zoonotic parasites are typically given little consideration during the implementation of mass deworming efforts. Accordingly, increased awareness of the potential impact of *A*. *ceylanicum* infection on the human population is required, and more sensitive tools are needed for monitoring this important zoonotic STH. Such tools will provide a more complete picture of the role such zoonotic parasites play in global health.

While current treatments for human hookworm infection are not species-dependent, it has been demonstrated that responses to drug treatments can vary in a species-dependent manner [30]. Because species-specific diagnostics are lacking, misidentification of the infecting species may lead to a lack of clarity in understanding drug-responses in infected populations. While benzimidazole-based interventions have not yet resulted in the reporting of any such species-specific responses, such responses may develop, or they may simply be underreported. As *A*. *duodenale* and *A*. *ceylanicum* are both capable of causing significant human infection, and *A*. *caninum* has the capacity to infect humans in an “unsuccessful” manner [23, 31], species-specific diagnostic assays are needed to fully evaluate the progress of drug intervention programs. Furthermore, given the increasing concerns surrounding the possible development of drug resistance [32–34], species-level knowledge of infection is critical, as it is likely that different hookworm species may evolve resistance *via* different mechanisms and at different rates. The importance of species-level identification is further supported by the phenomenon of *refugia*, as the presence of an untreated animal reservoir may slow the evolutionary pressures on a pathogen, in turn slowing the development of drug resistance mechanisms [32, 34].

While the *A*. *ceylanicum*-specific assay described here has potential usefulness as a tool for monitoring veterinary infections, its specificity and performance in such settings has not yet been evaluated. In order to evaluate such applications, additional testing and validation of this assay against common animal hookworm species such as *Ancylostoma braziliense*, *Ancylostoma tubaeforme*, and *Uncinaria stenocephala*, as well as other common veterinary intestinal helminths would be required. Similarly, employment of this assay for the purposes of environmental monitoring would require, at a minimum, testing against common free-living nematode species.

Given the importance of the accurate, species-level identification of STH infections, we believe that the real-time PCR-based assay for the detection of *A*. *ceylanicum* described here, in combination with our previously described real-time PCR assays, will aid future hookworm monitoring efforts. With the demonstrated capacity to detect DNA isolated from a single egg, this assay provides a sensitive and species-specific diagnostic tool capable of more fully informing program managers, enabling more appropriate decision-making and allowing for improved programmatic outcomes. Finally, it should be noted that this *A*. *ceylanicum* PCR assay is easily integrated into our current multi-parallel PCR system [25] to provide a more complete picture of the STH infection status in studied populations.

## Supporting information

S1 Fig *Ancylostoma* spp. determination on field samples from Orán, Argentina using semi-nested PCR-RFLP analysis. The testing was performed in the same manner as described in Fig 1 (main manuscript). The banding pattern demonstrates the presence of *A*. *duodenale* in these samples and validates the negative results from the newly described real-time PCR assay for *A*. *ceylanicum*.
(TIF)

S1 Table *Ancylostoma* spp. mixed infections and average Ct values from real-time PCR testing for *A*. *ceylanicum*. (XLSX)

S2 Table Average Ct values from real-time PCR testing for *A. duodenale* and *A. ceylanicum* with samples from Timor-Leste. DNA extracts from Timor-Leste were tested from two separate laboratories (QIMR, Australia and Smith College, USA) in duplicate using real-time PCR assays specific for *Ancylostoma duodenale* and *Ancylostoma ceylanicum*. The average Ct values from the assays are shown in this table; "-" indicates that infection was not present.
(XLSX)

S3 Table Average Ct values from real-time PCR testing for *A*. *duodenale* and *A*. *ceylanicum* with samples from Orán, Argentina. DNA extracts from Orán, Argentina, were tested from two separate laboratories (Baylor College of Medicine (BCM), USA and Smith College, USA) in duplicate using real-time PCR assays specific for *A*. *duodenale* and *A*. *ceylanicum*. The average Ct values from the testing are shown in this table; "-" indicates that infection was not present.
(XLSX)

## References

[1] Ngui R, Lim YAL, Ismail WHW, Lim KN, Mahmud R. Zoonotic *Ancylostoma ceylanicum* infection detected by endoscopy. Am J Trop Med Hyg. 2014; 91(1): 86–88. doi: 10.4269/ajtmh.13-0756 24891471 10.4269/ajtmh.13-0756 PMC4080576

[2] Bradbury RS, Hii SF, Harrington H, Speare R, Traub R. *Ancylostoma ceylanicum* hookworm in the Solomon Islands. Emerg Infect Dis. 2017; 23(2): 252–257. doi: 10.3201/eid2302.160822 28098526 10.3201/eid2302.160822 PMC5324822

[3] Hu W, Wu S, Yu X, Abullahi AY, Song M, Tan L, et al A multiplex PCR for simultaneous detection of three zoonotic parasites *Ancylostoma ceylanicum*, *Ancylostoma caninum*, and *Giardia lamblia* assemblage A. BioMed Res Int. 2015; 2015:406168 doi: 10.1155/2015/406168 26447336 10.1155/2015/406168 PMC4568324

[4] Traub RJ, Inpankaew T, Sutthikornchai C, Sukthana Y, Thompson RCA. PCR-based coprodiagnostic tools reveal dogs as reservoirs of zoonotic ancylostomiasis caused by *Ancylostoma ceylanicum* in temple communities in Bangkok. Vet Parasitol. 2008; 155(1–2): 67–73. doi: 10.1016/j.vetpar.2008.05.001 18556131 10.1016/j.vetpar.2008.05.001

[5] Ngui R, Lim YAL, Traub RJ, Mahmud R, Mistam MS. Epidemiological and genetic data supporting the transmission of *Ancylostoma ceylanicum* among human and domestic animals. PLoS Negl Trop Dis. 2012; 6(2): e1522 doi: 10.1371/journal.pntd.0001522 22347515 10.1371/journal.pntd.0001522 PMC3274503

[6] Inpankaew T, Schär F, Dalsgaard A, Khieu V, Chimnoi W, Chhoun C, et al High prevalence of *Ancylostoma ceylanicum* hookworm infections in humans, Cambodia, 2012. Emerg Infect Dis. 2014; 20(6): 976–982. doi: 10.3201/eid2006.131770 24865815 10.3201/eid2006.131770 PMC4036766

[7] Conlan JV, Khamlome B, Vongxay K, Elliot A, Pallant L, Sripa B, et al Soil-transmitted helminthiasis in Laos: a community-wide cross-sectional study of humans and dogs in a mass drug administration environment. Am J Trop Med Hyg. 2012; 86(4): 624–634. doi: 10.4269/ajtmh.2012.11-0413 22492147 10.4269/ajtmh.2012.11-0413 PMC3403769

[8] Jiraanankul V, Aphijirawat W, Mungthin M, Khositnithikul R, Rangsin R, Traub RJ, et al Incidence and risk factors of hookworm infection in a rural community of central Thailand. Am J Trop Med Hyg. 2011; 84(4): 594–598. doi: 10.4269/ajtmh.2011.10-0189 21460016 10.4269/ajtmh.2011.10-0189 PMC3062455

[9] Baker MK, Lange L, Verster A, van der Plaat S. A survey of helminths in domestic cats in the Pretoria area of Transvaal, Republic of South Africa. Part 1: The prevalence and comparison of burdens of helminths in adult and juvenile cats. J S Afr Vet Assoc. 1989; 60(3): 139–142. 2634770

[10] Rep BH, Heinemann DW. Changes in hookworm distribution in Surinam. Trop Geogr Med. 1976; 28(2): 104–110. 987625

[11] Schuster RK, Thomas K, Sivakumar S, O’Donovan D. The parasite fauna of stray domestic cats (*Felis catus*) in Dubai, United Arab Emirates. Parasitol Res. 2009; 105(1): 125–134. doi: 10.1007/s00436-009-1372-6 19238440 10.1007/s00436-009-1372-6

[12] Anten JF, Zuidema PJ. Hookworm infection in Dutch servicemen returning from West New Guinea. Trop Geogr Med. 1964; 16: 216–224. 5895548

[13] Palmer CS, Traub RJ, Robertson ID, Hobbs RP, Elliot A, While L, et al The veterinary and public health significance of hookworm in dogs and cats in Australia and the status of *A*. *ceylanicum*. Vet Parasitol. 2007; 145(3–4): 304–313. doi: 10.1016/j.vetpar.2006.12.018 17276602 10.1016/j.vetpar.2006.12.018

[14] Scholz T, Uhlírová M, Ditrich O. Helminth parasites of cats from the Vientiane province, Laos, as indicators of the occurrence of causative agents of human parasitoses. Parasite. 2003; 10(4): 343–350. doi: 10.1051/parasite/2003104343 14710631 10.1051/parasite/2003104343

[15] Traub RJ. *Ancylostoma ceylanicum*, a re-emerging but neglected parasitic zoonosis. Int J Parasitol. 2013; 43(12–13): 1009–1015. doi: 10.1016/j.ijpara.2013.07.006 23968813 10.1016/j.ijpara.2013.07.006

[16] Setasuban P, Vajrasthira S, Muennoo C. Prevalence and zoonotic potential of *Ancylostoma ceylanicum* in cats in Thailand. Southeast Asian J Trop Med Public Health. 1976; 7(4): 534–539. 1030851

[17] Albonico M, Stoltzfus RJ, Savioli L, Tielsch JM, Chwaya HM. Epidemiological evidence for a different effect of hookworm species, *Ancylostoma duodenale* or *Necator americanus*, on iron status of children. Int J Epidemiol 1998; 27 (3): 530–537. 9698148 10.1093/ije/27.3.530

[18] Jonker FAM, Calis JCJ, Phiri K, Brienen EAT, Khoffi H, Brabin BJ, et al Real-time PCR demonstrates *Ancylostoma duodenale* is a key factor in the etiology of severe anemia and iron deficiency in Malawian pre-school children. PLoS Negl Trop Dis. 2012; 6(3): e1555 doi: 10.1371/journal.pntd.0001555 22514750 10.1371/journal.pntd.0001555 PMC3295794

[19] Crompton DW. The public health importance of hookworm disease. Parasitology. 2000; 121 Suppl: S39–S50. 11386690 10.1017/s0031182000006454

[20] Lucio-Forster A, Liotta JL, Yaros JP, Briggs KR, Mohammed HO, Bowman DD. Morphological differentiation of eggs of *Ancylostoma caninum*, *Ancylostoma tubaeforme*, and *Ancylostoma braziliense* from dogs and cats in the United States. J Parasitol. 2012; 98(5): 1041–1044. doi: 10.1645/GE-2928.1 22394087 10.1645/GE-2928.1

[21] de Gruijter JM, van Lieshout L, Gasser RB, Verweij JJ, Brienen EAT, Ziem JB, et al Polymerase chain reaction-based differential diagnosis of *Ancylostoma duodenale* and *Necator americanus* infections in humans in northern Ghana. Trop Med Int Health. 2005;10(6): 574–580. doi: 10.1111/j.1365-3156.2005.01440.x 15941421 10.1111/j.1365-3156.2005.01440.x

[22] Yamamoto Y. PCR in diagnosis of Infection: detection of bacteria in cerebrospinal fluids. Clin Diagn Lab Immunol. 2002; 9(3): 508–514. doi: 10.1128/CDLI.9.3.508-514.2002 11986253 10.1128/CDLI.9.3.508-514.2002 PMC119969

[23] George S, Kaliappan SP, Kattula D, Roy S, Geldhof P, Kang G, et al Identification of *Ancylostoma ceylanicum* in children from a tribal community in Tamil Nadu, India using a semi-nested PCR-RFLP tool. Trans R Soc Trop Med Hyg. 2015; 109(4): 283–285. doi: 10.1093/trstmh/trv001 25618132 10.1093/trstmh/trv001

[24] Traub RJ, Robertson ID, Irwin P, Mencke N, Thompson RCA. Application of a species-specific PCR-RFLP to identify *Ancylostoma* eggs directly from canine faeces. Vet Parasitol. 2004; 123(3–4): 245–255. doi: 10.1016/j.vetpar.2004.05.026 15325050 10.1016/j.vetpar.2004.05.026

[25] Pilotte N, Papaiakovou M, Grant JR, Bierwert LA, Llewellyn S, McCarthy JS, et al Improved PCR-based detection of soil transmitted helminth infections using a next-generation sequencing approach to assay design. PLoS Negl Trop Dis. 2016; 10(4): e0004578. 27027771 10.1371/journal.pntd.0004578 PMC4814118

[26] Novák P, Neumann P, Pech J, Steinhaisl J, Macas J. RepeatExplorer: a Galaxy-based web server for genome-wide characterization of eukaryotic repetitive elements from next-generation sequence reads. Bioinformatics. 2013; 29(6): 792–793. doi: 10.1093/bioinformatics/btt054 23376349 10.1093/bioinformatics/btt054

[27] Llewellyn S, Inpankaew T, Nery SV, Gray DJ, Verweij JJ, Clements ACA, et al Application of a multiplex quantitative PCR to assess prevalence and intensity of intestinal parasite infections in a controlled clinical trial. PLoS Negl Trop Dis. 2016; 10(1): e0004380 doi: 10.1371/journal.pntd.0004380 26820626 10.1371/journal.pntd.0004380 PMC4731196

[28] Cimino RO, Jeun R, Juarez M, Cajal PS, Vargas P, Echazú A, et al Identification of human intestinal parasites affecting an asymptomatic peri-urban Argentinian population using multi-parallel quantitative real-time polymerase chain reaction. Parasit Vectors. 2015; 8:380 doi: 10.1186/s13071-015-0994-z 26183074 10.1186/s13071-015-0994-z PMC4504406

[29] Schwarz EM, Hu Y, Antoshechkin I, Miller MM, Sternberg PW, Aroian RV. The genome and transcriptome of the zoonotic hookworm *Ancylostoma ceylanicum* identify infection-specific gene families. Nat genet. 2015; 47(4): 416–422. doi: 10.1038/ng.3237 25730766 10.1038/ng.3237 PMC4617383

[30] Reynoldson JA, Behnke JM, Pallant LJ, Macnish MG, Gilbert F, Giles S, et al Failure of pyrantel in treatment of human hookworm infections (*Ancylostoma duodenale*) in the Kimberley region of north west Australia. Acta Trop. 1997; 68(3): 301–312. 9492915 10.1016/s0001-706x(97)00106-x

[31] Landmann JK, Prociv P. Experimental human infection with the dog hookworm, *Ancylostoma caninum*. Med J Aust. 2003; 178(2): 69–71. 12526725 10.5694/j.1326-5377.2003.tb05222.x

[32] Vercruysse J, Albonico M, Behnke JM, Kotze AC, Prichard RK, McCarthy JS, et al Is anthelminthic resistance a concern for the control of human soil-transmitted helminths? Int J Parasitol Drugs Drug Resist. 2011; 1(1): 14–27. doi: 10.1016/j.ijpddr.2011.09.002 24533260 10.1016/j.ijpddr.2011.09.002 PMC3913213

[33] Vercruysse J, Levecke B, Prichard R. Human soil-transmitted helminths: implications of mass drug administration. Curr Opin Infect Dis. 2012; 25(6): 703–708. doi: 10.1097/QCO.0b013e328358993a 22964945 10.1097/QCO.0b013e328358993a

[34] Shalaby HA. Anthelmintics Resistance; How to Overcome it? Iran J Parasitol. 2013; 8(1): 18–32. 23682256 PMC3655236

