# Supplementary figures and images for: A novel, species-specific, real-time PCR assay for the detection of the emerging zoonotic parasite Ancylostoma ceylanicum in human stool
Source: PLoS Negl Trop Dis. 2017 Jul 10;11(7):e0005734. doi: 10.1371/journal.pntd.0005734 (PMC5519186; doi:10.1371/journal.pntd.0005734)

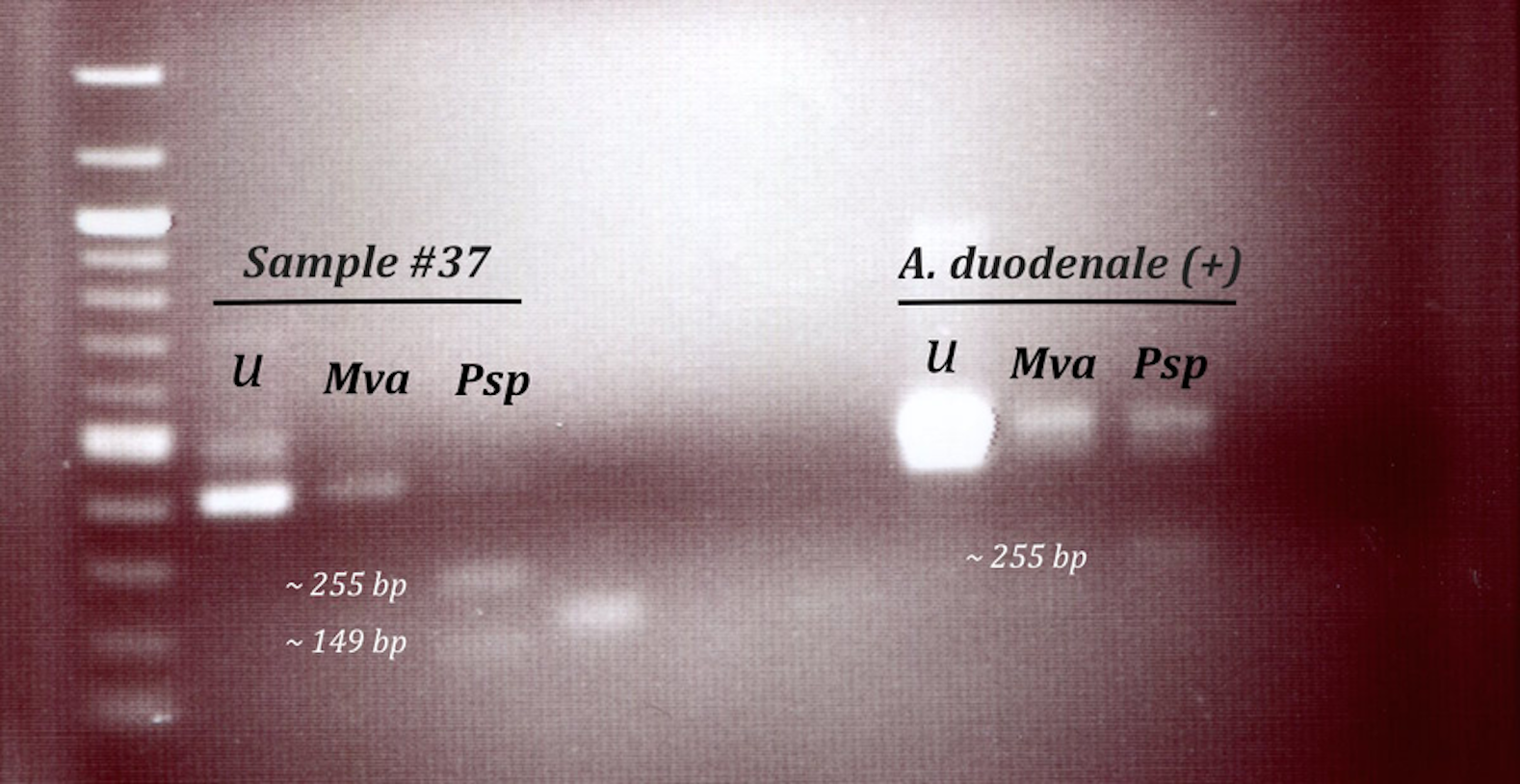

Supplement: S1 Fig — The testing was performed in the same manner as described in Fig 1 (main manuscript). The banding pattern demonstrates the presence of A. duodenale in these samples and validates the negative results from the newly described real-time PCR assay for A. ceylanicum. (TIF) [file pntd.0005734.s001.tif]
